# Association of Living Alone and Same-Day Discharge After Hysterectomy for Benign Gynecologic Indications

**DOI:** 10.1097/og9.0000000000000122

**Published:** 2025-10-09

**Authors:** Abdelrahman Yousif, Mohanad Elchouemi, Brandon Godinich, Sarah Johnson

**Affiliations:** Department of Obstetrics and Gynecology and the Paul L. Foster School of Medicine, Texas Tech Health El Paso, El Paso, Texas.

## Abstract

**OBJECTIVE:**

To evaluate the association between social support status (living alone vs living with others) and same-day discharge rates among patients undergoing hysterectomy for benign indications.

**METHODS:**

We conducted a retrospective cohort study that used data from the American College of Surgeons National Surgical Quality Improvement Program 2021–2022 Participant Use File. Patients undergoing hysterectomy for benign indications were identified based on Current Procedural Terminology and International Classification of Diseases, Tenth Revision codes. The primary exposure was social support status, categorized as “Lives Alone” or “Lives with Others”; the primary outcome was *same-day discharge*, defined as discharge on the calendar day of surgery. Covariates included age, body mass index (BMI), race, ethnicity, diabetes, hypertension, surgical approach, and operative time. Multivariable logistic regression was performed to assess the association between living alone and same-day discharge, adjusting for relevant clinical and demographic factors.

**RESULTS:**

A total of 1,615 patients were included, with 30% living alone and 70% living with others. Overall, 26% of patients had same-day discharge. Same-day discharge occured less frequently among patients living alone (22%) compared with those living with others (28%) (odds ratio [OR] 1.3; *P=*.02; CI, 1.0–1.7). In multivariable analysis, living alone was associated with 32% higher odds of overnight stay compared with living with others (OR 1.3; 95% CI, 1.0–1.7; *P*=.047). Surgical approach was a strong predictor of discharge outcomes, with laparoscopic hysterectomy (OR 0.4; 95% CI, 0.3–0.6) associated with reduced odds of overnight stay compared with vaginal hysterectomy. Operative time more than 180 minutes was associated with a nonsignificant trend towards increased overnight stay (OR 1.2; 95% CI, 0.8–1.5).

**CONCLUSION:**

Living alone is associated with lower odds of same-day discharge after hysterectomy for benign indications, independent of clinical and demographic characteristics. These findings underscore the need to consider social support during preoperative planning to optimize discharge outcomes. Strategies to enhance home support may facilitate same-day discharge among patients living alone.

## Abstract 2

Living alone is associated with reduced same-day discharge after hysterectomy for benign indications, underscoring the importance of social support in discharge planning.

By 2030, one in six individuals worldwide will be aged 60 years or older, and the rate of population aging is accelerating, especially in low- and middle-income countries.^1^ In the United States, approximately 17% of adults older than age 65 years are hospitalized each year, compared with 8% of adults aged 45–64 years.^2^ As the surgical population ages, there is a growing need to understand factors that influence postoperative recovery and discharge planning, including the availability of social support at home. This demographic shift underscores the need for health systems to adapt to the complex care needs of older surgical patients.

Social support is recognized as a critical determinant of postoperative outcomes across various surgical specialties.^3–7^ Living alone may complicate postoperative self-care, mobility, and access to assistance, which can influence clinicians' discharge decisions.^8^ After hospitalization, recovery often requires multiple transitions of care, with informal or unpaid family caregivers frequently assuming responsibility for in-home care tasks and health care coordination.^9^ In the absence of such support, patients living alone may encounter greater barriers to safe recovery after surgery.

Same-day discharge after hysterectomy for benign indications has become increasingly common, driven by advances in minimally invasive surgical techniques, enhanced recovery protocols, and efforts to optimize hospital resource utilization.^10–12^ Prior studies have identified several predictors of same-day discharge, including age, comorbidities, and surgical approach.^13^ However, these factors largely reflect clinical variables, and less is known about the effect of social determinants such as home support availability.

Understanding whether living alone affects the likelihood of same-day discharge after hysterectomy for benign indications has important implications for preoperative counseling, risk stratification, and discharge planning. Therefore, we aimed to evaluate the association between social support status (living alone vs living with others) and same-day discharge rates among patients undergoing hysterectomy for benign indications. We hypothesized that patients living alone would have lower odds of same-day discharge compared with those with available support at home.

## METHODS

We conducted a retrospective cohort study using data from the American College of Surgeons National Surgical Quality Improvement Program (ACS NSQIP) 2021–2022 Participant Use File. The ACS NSQIP is a nationally validated, prospectively collected database that captures perioperative variables and 30-day postoperative outcomes across participating institutions in the United States. IRB approval was not required for this study because it used publicly available, deidentified data from the ACS NSQIP, and did not involve human subject interaction or identifiable private information. We identified patients who underwent hysterectomy for benign gynecologic indications based on Current Procedural Terminology and International Classification of Diseases, Tenth Revision codes (Appendix 1, available online at http://links.lww.com/AOG/E354). Exclusion criteria included procedures performed for malignant indications (as defined by disseminated cancer status or postoperative diagnosis codes), missing or unknown social support status, and missing discharge data. Benign gynecologic indications included abnormal uterine bleeding, leiomyomas, endometriosis, pelvic organ prolapse, benign neoplasms, and pelvic pain.

The primary exposure of interest was social support status, identified by the ACS NSQIP variable “HOMESUP,” which indicates whether the patient lived alone or with others at the time of surgery. Patients were categorized as “Lives Alone” or “Lives with Others.” Although this variable provides information on living arrangement, it does not capture the entire adequacy of the functional support provided to the patient and, thus, may oversimplify the construct of social support.

The primary outcome was *overnight stay*, defined as hospitalization extending beyond the calendar day of surgery. In contrast, same-day discharge was determined by the number of days from the operation to discharge, with a recorded duration of 0 days indicating discharge on the same day as surgery. Length of stay was quantified by calendar date, in accordance with ACS NSQIP definitions, rather than by elapsed hours.

The following patient- and surgery-related variables were included as covariates based on clinical relevance and review of the literature: age (continuous, in years), body mass index (BMI, calculated as weight in kilograms divided by height in meters squared, continuous), race (White vs non-White), ethnicity (Hispanic vs non-Hispanic), diabetes mellitus (yes or no), functional dependence, American Society of Anesthesiologists (ASA) classification, history of falls within the past 6 months, cognitive impairment, hypertension requiring medication (yes or no), surgical approach (abdominal, laparoscopic, laparoscopically assisted vaginal hysterectomy, or vaginal, with abdominal hysterectomy as the reference group), and operative time (categorized as less than 180 minutes vs 180 minutes or longer).

Descriptive statistics were used to compare baseline characteristics between patients living alone and those living with others. Continuous variables were reported as means and standard deviations and were compared using *t* tests or Wilcoxon rank sum tests, as appropriate. Categorical variables were compared using χ^2^ tests.

We performed multivariable logistic regression to assess the association between living alone and same-day discharge, adjusting for covariates selected a priori based on clinical relevance and prior literature. The final model included age, BMI, race, ethnicity, diabetes, hypertension (use of antihypertensive medications), surgical approach (laparoscopically assisted vaginal hysterectomy, laparoscopic, and vaginal), and operative time (more than 180 minutes). American Society of Anesthesiologists classification was considered initially; however, more than 95% of patients were classified as ASA II or III, and its inclusion did not meaningfully alter effect estimates. For parsimony and to minimize overfitting, ASA classification was omitted from the final model. Because 99% of patients (140/141) who underwent abdominal hysterectomy had an overnight stay, this group was excluded from all regression analyses to avoid sparse data bias (Appendix 2, available online at http://links.lww.com/AOG/E354). Race was included as a covariate, given evidence of racial disparities in surgical outcomes and health care utilization, ensuring adjustment for potential confounding.

Missing data for race (approximately 24%) and ethnicity (approximately 21%) were handled using multiple imputation with chained equations. Twenty imputed datasets were generated, with all covariates from the final model included as predictors in the imputation models. Regression estimates from each imputed dataset were pooled using Rubin's rules, and these pooled estimates are presented as the primary results.

Model performance was evaluated using the area under the receiver operating characteristic curve (AUROC). The AUROC was calculated for each imputed dataset, and the mean AUROC was reported along with the range across the 20 imputations. Statistical significance was defined as a two-tailed *P*<.05. All analyses were performed using Stata 18.0.

## RESULTS

A total of 1,615 patients undergoing hysterectomy for benign indications were identified and included in the baseline analysis. Among them, 483 patients (30%) lived alone and 1,132 (70%) lived with others. Overall, 419 patients were discharged on the same day as surgery.

Baseline characteristics stratified by social support status are shown in Table 1. Patients who lived alone were slightly older than those living with others (mean age 79 years vs 78 years, *P*<.001) and were less likely to be of non-White racial backgrounds (*P*=.008). No significant differences were observed in rates of diabetes mellitus, hypertension, smoking status, functional dependence, history of falls, or cognitive impairment. Surgical approach and surgical indication were also comparable between groups, with lower rates of same-day discharge observed among those living alone (22% vs 28%; 95% CI, 1.0–1.7; *P*=.01).

**Table 1.** Baseline Patient Characteristics Stratified by Social Support Status

Characteristic	Lives Alone	Lives With Others	*P*
Total	483 (30)	1,132 (70)	
Age (y)	79±4	78±3	**<**.001
Ethnicity			.07
Hispanic	27 (6)	95 (8)	
Non-Hispanic	344 (71)	803 (71)	
Missing	112 (23)	234 (21)	
Race			.008
Non-White*	33 (7)	130 (11)	
White	323 (67)	742 (65)	
Missing	127 (26)	260 (23)	
BMI (kg/m^2^)	28±6	27±5	.005
Diabetes mellitus	68 (14)	190 (17)	.2
Hypertension, on medication	313 (65)	750 (66)	.6
Current smoking	17 (4)	31 (3)	.4
ASA classification			.2
I	7 (1)	7 (1)	
II	227 (47)	554 (49)	
III	242 (50)	548 (48)	
IV	6 (1)	21 (2)	
Functional status before surgery			.05
Independent	478 (99)	1,097 (97)	
Partially dependent	3 (0.6)	19 (2)	
Unknown	2 (0.4)	16 (1)	
History of fall within 6 mo	17 (4)	31 (3)	.3
History of cognitive impairment	11 (2)	37 (3)	.3
Same-day discharge			.01
Yes	106 (22)	313 (28)	
No	377 (78)	819 (72)	
Operative time (min)			
Less than 180	371 (77)	852 (75)	.5
180 or more	112 (23)	280 (25)	
Blood transfusion	11 (2)	12 (1)	.05
Surgical approach			.4
Laparoscopic	149 (31)	342 (30)	
Abdominal	49 (10)	92 (8)	
Vaginal	239 (49)	596 (53)	
LAVH	46 (10)	102 (9)	
Benign indication			.05
POP	296 (61)	776 (69)	
Uterine leiomyomas	61 (13)	109 (10)	
Benign ovarian cysts and neoplasm	81 (17)	174 (15)	
Abnormal menstruation	33 (7)	56 (5)	
Endometriosis	11 (2)	16 (1)	
Pelvic pain	0 (0)	1 (0.1)	
Dysmenorrhea	1 (0.2)	0 (0)	

BMI, body mass index; ASA, American Society of Anesthesiologists; LAVH, laparoscopic assisted vaginal hysterectomy; POP, pelvic organ prolapse.

Data are mean±SD or n (%) unless otherwise specified.

* Includes American Indian, Alaska Native, Asian, Black or African American, Native Hawaiian, and Pacific Islander.

After excluding patients who underwent abdominal hysterectomy (99% of whom had overnight stays), 1,464 patients were included in the multiple imputation analysis (Table 2). Living alone was significantly associated with increased odds of overnight stay compared with living with others (odds ratio [OR] 1.3; 95% CI, 1.0–1.7; *P*=.047). Age also was an independent predictor, with each additional year increasing the odds of overnight admission by 6% (OR 1.1; 95% CI, 1.0–1.1; *P*=.002). Body mass index, race, Hispanic ethnicity, diabetes, and antihypertensive use were not significantly associated with the outcome.

**Table 2.** Factors Associated With Overnight Stay

Factor	Adjusted OR (95% CI)*	*P*
Social support status		
Lives with others (ref)	—	—
Lives alone	1.3 (1.0–1.7)	.047
Age (per year increase)	1.1 (1.0–1.1)	.002
Surgical approach		
Vaginal (ref)	—	—
Laparoscopic	0.4 (0.3–0.6)	<.001
LAVH	1.2 (0.7–1.8)	.4
Operative time (min)		
Less than 180 (ref)	—	—
180 or more	1.2 (0.8–1.5)	.2
BMI	1.0 (0.9–1.0)	.4
Race		
Non-White	0.9 (0.6–1.4)	.8
White (ref)	—	—
Ethnicity		
Hispanic	0.6 (0.4–1)	.07
Non-Hispanic (ref)	—	—
Diabetes		
No (ref)	—	—
Yes	1.1 (0.8–1.6)	.3
Hypertension	—	—
No (ref)		
Yes	0.8 (0.7–1.1)	.3

OR, odds ratio; ref, referent; LAVH, laparoscopic assisted vaginal hysterectomy; BMI, body mass index.

* Adjusted for the following covariates: age, BMI, race, ethnicity, diabetes, hypertension, surgical approach, and operative time. Results based on multiple imputation. The average area under the receiver operating characteristic curve across the 20 imputed datasets was 0.634 (range 0.632–0.637), indicating modest discrimination.

Among surgical approaches, laparoscopic procedures were associated with significantly lower odds of an overnight stay compared with vaginal hysterectomy (OR 0.4; 95% CI, 0.3–0.6; *P*<.001), whereas laparoscopically assisted vaginal hysterectomy showed no significant difference from vaginal hysterectomy (OR 1.2; 95% CI, 0.7–1.8; *P*=.4). An operative time of more than 180 minutes was not significantly associated with the outcome (OR 1.2; 95% CI, 0.8–1.5; *P*=.2). The AUROC across the 20 imputed datasets was 0.634 (range, 0.6–0.6), indicating modest discrimination.

## DISCUSSION

In this retrospective cohort study of patients undergoing hysterectomy for benign indications, we found that living alone was associated with higher odds of overnight stay compared with living with others, even after adjusting for age, BMI, race, ethnicity, diabetes, hypertension, surgical approach, and operative time. Increasing age also was independently associated with higher odds of an overnight stay. As expected, laparoscopic hysterectomy was strongly associated with successful same-day discharge, demonstrating significantly lower odds of overnight stay compared with vaginal approach in our study cohort.

Although living alone was associated with higher odds of overnight stay and reached statistical significance (OR 1.3; 95% CI, 1.0–1.7; *P*=.047), the effect size was modest. This finding should be interpreted with caution, because statistical significance often does not equate to clinical significance. Nonetheless, even modest associations may have meaningful implications at the population level, particularly for discharge and planning resource allocation.

Our findings align with a broader body of literature demonstrating the critical role of social support in postoperative recovery across different surgical populations.^7,14–16^ Studies in older adult trauma patients have highlighted that after discharge, many individuals experience decreased mobility and greater reliance on informal caregivers for assistance with daily tasks and medical management.^17–19^ Adequate caregiver support facilitates recovery, whereas its absence may contribute to delayed discharge and higher readmission risk.^20^

Similarly, research examining interventions to facilitate earlier hospital discharge in older adults suggests that social context—including living arrangements—plays a key role in discharge decision-making.^21,22^ Programs aimed at enhancing home support, such as assistive technologies and structured rehabilitation services, have been associated with reduced caregiver burden and improved patient outcomes.^23,24^ Such strategies could have adapted to support patients identified as living alone in the preoperative period. These findings further underscore the relevance of evaluating social determinants such as living situation when considering eligibility for same-day discharge after gynecologic surgeries for benign indications. A safe disposition goes beyond a stable place to live and recover; it also necessitates adequate support throughout the recovery process.

The strong association between minimally invasive surgical approaches and same-day discharge observed in our study is consistent with established evidence. Minimally invasive techniques are associated with faster recovery times, reduced pain, and fewer perioperative complications compared with open surgery.^25,26^ Although the magnitude of the ORs for the surgical approach was large, this likely reflects the well-established clinical benefits of less invasive approaches on discharge readiness, rather than an inherent bias.

Strengths of our study include the use of a large, contemporary cohort, rigorous covariate adjustment based on clinical relevance and focus on a modifiable factor—social support—that has not been previously studied in the context of hysterectomy discharge outcomes. By highlighting the potential vulnerability of patients living alone, our findings may inform preoperative counseling, postoperative planning, and the development of supportive interventions tailored to social circumstances. Another strength is the use of a national surgical registry, increasing generalizability.

Limitations should be acknowledged. First, social support was inferred from the ACS NSQIP's “HOMESUP” variable, which consisted of two options: living alone or with others. The variable is limited in that it oversimplifies patients' support systems and does not directly measure functional support at home (eg, family, community, or health aide assistance). Although living alone is used as a proxy for low social support in our study, it may not fully capture the complexity of a patient's support network, as some individuals living alone may still have substantial community or familial support that influences postoperative recovery. Second, although we adjusted for key clinical and surgical variables, residual confounding may still exist. Third, our study cohort was restricted to patients undergoing hysterectomy for benign indications, thereby limiting applicability to oncologic or emergent hysterectomy procedures, which typically require more extensive postoperative care. In addition, ACS NSQIP–participating centers are primarily large, urban, and academic hospitals, and represent a voluntary network of institutions committed to quality improvement. As a result, the findings may not be generalizable to all hospital settings. We acknowledge that the absence of data on hospital characteristics and insurance status may contribute to unmeasured confounding. These factors could influence discharge practices and access to home support services, and their omission is a limitation of our study. Finally, given the retrospective nature of the dataset, unmeasured confounding variables cannot be fully accounted for, and causal inferences should be made cautiously.

In conclusion, living alone was independently associated with higher odds of overnight stay after hysterectomy for benign indications, after adjustment for demographic, clinical, and surgical factors. As health care systems increasingly prioritize same-day discharge models, our findings underscore the importance of systematically assessing social support during preoperative planning.

Social support is a potentially modifiable risk factor for same-day discharge eligibility. Incorporating targeted interventions—such as routine preoperative screening for home support, structured discharge planning, telehealth follow-up, and engagement of home health aides—could help identify and address barriers for patients at risk of delayed discharge. Implementing these measures within enhanced recovery protocols may enable more patients living alone to safely benefit from same-day discharge pathways.

Future research should prospectively evaluate the effectiveness of such interventions, including community-based assistance programs, in optimizing discharge outcomes for patients lacking robust home support. Policy efforts to integrate social support assessment and mitigation strategies into perioperative care could facilitate more equitable access to same-day discharge and efficient use of health care resources.
